# Paclitaxel disrupts polarized entry of membrane-permeable C6 ceramide into ovarian cancer cells resulting in synchronous induction of cell death

**DOI:** 10.3892/ol.2013.1305

**Published:** 2013-04-16

**Authors:** CHARLES BEST, DAVID CALIANESE, KEVIN SZULAK, GARRET CAMMARATA, GABRIELLA BRUM, THOMAS CARBONE, ERIC STILL, KATELYN HIGGINS, FANG JI, WEN DI, HAROLD WANEBO, YINSHENG WAN

**Affiliations:** 1 Department of Biology, Providence College, Providence, RI 02918, USA;; 2 Department of Obstetrics and Gynecology, Renji Hospital of Shanghai Jiaotong University Medical School, Shanghai 200001, P.R. China;; 3 Department of Surgery, Landmark Medical Center, Woonsocket, RI 02895, USA

**Keywords:** C6 ceramide, AKT, ovarian cancer, paclitaxel

## Abstract

Exogenous cell-permeable C6 ceramide has been demonstrated to act synergistically with chemotherapeutic drugs, including paclitaxel, cisplatin, doxorubicin and the histone deacetylase inhibitor, trichostatin A, to induce cell death in a variety of cancer cells. We previously demonstrated that C6 ceramide and paclitaxel function synergistically to induce ovarian cancer cell death via modulation of the PI3/AKT cell survival pathway. In the present study, the entry pattern of C6 ceramide into ovarian cancer cells was investigated using fluorescent short chain C6-NBD sphingomyelin (C6-NBD). Confocal microscopy revealed that C6-NBD enters the cells in a polarized pattern, characterized by marked signals at one cellular end, representing a likely mitosis initiation site. Pretreatment of the cells with filipin, an inhibitor of the lipid raft/caveolae endocytosis pathway, decreases C6-NBD entry into the cells. A pretreatment with the water channel inhibitor, CuSO_4_, was also found to reduce the entry of C6-NBD. Notably, the pretreatment with paclitaxel was shown to disrupt the polarized entry of C6-NBD into the cells, resulting in an even distribution of C6-NBD in the cytoplasm. In addition, the pretreatment of the cells with paclitaxel destabilized the cytoskeletal proteins, releasing an increased number of short tubulin fragments. The results of the present study indicate that C6 ceramide preferentially enters the cells via a predetermined initiation site of mitosis. In addition to diffusion, short chain C6 ceramide may also enter cells via water channels and caveolae-mediated endocytosis. Paclitaxel disrupts the cell cytoskeleton and induces an even distribution of C6 ceramide in the cytoplasm resulting in synergistic ovarian cancer cell death.

## Introduction

The function of ceramide in apoptosis and its association with receptor-associated apoptotic signaling proteins remain unresolved. It has previously been shown that TNF-α-induced apoptosis is preceded by an increase in intracellular ceramide levels (1). TNF-α and exogenous C6 ceramide interfere with the activation of Raf-1 and ERK by EGF and down-regulate v-Src-induced Raf-1 kinase activity (1). Exogenous C6 ceramide induces endocytic vesicle formation and results in enlarged late endosomes and lysosomes in mouse fibroblasts (2).

Chemotherapeutic agents, including paclitaxel and taxol, as well as physiological stimuli, such as TNF-α, stimulate ceramide accumulation and increase oxidative stress in cancer cells, and the upregulation of glucosylceramide synthase has been hypothesized to contribute to chemoresistance (3). Notably, multidrug-resistant cancer cells exhibit elevated levels of glucosylceramide (4–7). Agents that block ceramide glycosylation potentiate the cellular sensitivity to ceramide and chemotherapeutic agents, indicating that the ceramide metabolic pathway is an important target for anticancer drug development (8).

Paclitaxel has emerged as a valuable antimitotic chemotherapy drug, particularly in breast and ovarian cancer (9). Although cytotoxic mechanisms are well understood, the efficacy of this drug cannot be explained by microtubular interactions only. Paclitaxel-induced apoptosis has been shown to be attributable, in part, to ceramide and sphingoid bases, and the simultaneous treatment of Jurkat cells with paclitaxel and ceramide has been demonstrated to enhance paclitaxel-induced cell growth inhibition (10). Paclitaxel/ceramide combination therapy has been actively studied (11) and the clinical use of paclitaxel with ceramide-enhancing agents may maximize cytotoxic potential (12).

Our previous studies have demonstrated that the combination of paclitaxel and ceramide synergistically induced pancreatic cancer cell death through differential modulation of EGFR-mediated MAP kinases. EGFR and ERK inhibitors may further enhance the effect of paclitaxel and ceramide (13). The combination of paclitaxel and ceramide in biodegradable polymeric nanoparticles has been identified as an extremely effective therapeutic strategy to overcome drug resistance in ovarian cancer (14).

Additional studies have identified a ceramide transport protein, COL4A3BP or CERT, which sensitizes cancer cells to multiple cytotoxic agents when downregulated. COL4A3BP expression is increased in drug-resistant cell lines and in residual tumor cells following paclitaxel treatment of ovarian cancer, indicating that it may be a target for chemotherapy-resistant cancers (15–17).

Considering the rising functions of ceramide in combinatorial therapies with other chemotherapeutic agents, and the involvement of its modified form in chemoresistance, the entry of exogenous C6 ceramide was analyzed in the present study using fluorescently-labeled C6-NBD. C6 ceramide was observed to enter the ovarian cancer cells in a polarized fashion. In addition to this, paclitaxel was observed to induce vesicle formation and prevent the polarized entry of C6 into the cancer cells, thus exhibiting a synergistic effect on apoptosis.

## Materials and methods

### Chemicals and reagents

C6-NBD-ceramide was a gift from Avanti Polar Lipids, Inc. (Alabaster, AL, USA). Filipin, taxol and doxorubicin were obtained from Sigma-Aldrich (St. Louis, MO, USA). Hoechst 33342 was purchased from Molecular Probes (Calsbad, CA, USA).

### Cell culture

Human ovarian cancer cells (CaOV3 cells) were maintained as described previously (18) in DMEM (Sigma-Aldrich) supplemented with 10% fetal bovine serum, penicillin/streptomycin (1:100, Sigma-Aldrich) and 4 mM L-glutamine, in a CO_2_ incubator at 37°C.

### Confocal microscopy

The cells were plated in eight-well chamber slides (Lab-Tek; Nalge Nunc International, Naperville, IL, USA) and treated with various reagents, including C6-NBD ceramide, taxol and CuSO_4_. Next, the cells were either left untreated or were fixed for 20 min in fresh 4% paraformaldehyde-PBS. The cell nuclei were also stained with Hoechst (1 *μ*g/ml in PBS) for 10 min. The slides were mounted with anti-fade (Life Technologies, Grand Island, NY, USA) and stored in the dark until viewing. The samples were observed under a confocal microscope and images were captured by Zen 2009 Light Edition (Carl Zeiss AG, Oberkochen, Germany).

## Results and Discussion

### C6 ceramide enters cells in a polarized manner

**Figure 1:**
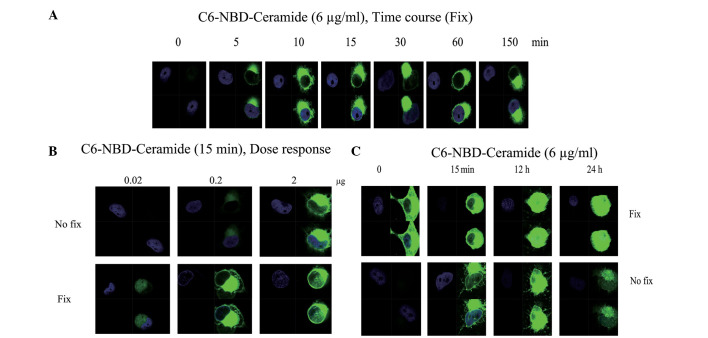
Polarized distribution of C6-NBD ceramide in ovarian cancer cells. (A and C) CaOV3 cells were cultured in 8-well chamber slides and treated with C6-NBD ceramide (6 *μ*g/ml). (B) The cells were fixed with formaldehyde at various times following treatment or were treated with the indicated doses of C6-NBD-ceramide, with or without fixation. The cells were processed and observed under a confocal microscope. Fluorescent short chain C6-NBD sphingomyelin, C6-NBD; Green, C6-NBD-ceramide; blue, nuclear staining.

The use of a combination of several chemotherapeutic agents is well accepted clinically, as it enables drugs to be administered at relatively low doses with an improved efficacy. Our previous studies demonstrated that C6 ceramide functions synergistically with taxol to inhibit cell proliferation and cell migration in cultured ovarian cancer cells (18). However, the molecular mechanism of this synergism remains unknown, and the entry of membrane-permeable C6 ceramide into the cells remains uncharacterized. To investigate the pattern of C6 entry into the cells, fluorescently-labeled C6-NBD was used. Ovarian cancer cells were treated with C6 ceramide and the resultant fluorescence signal was observed with or without fixation. The results indicated that C6 ceramide enters the cells in a time- (Fig. 1A) and dose-dependent (Fig. 1B) manner. Notably, the distribution of the fluorescence signal showed a polarized pattern (Fig. 1A and B). Subsequent to 12 h, the fluorescence signal had saturated the cells (Fig. 1C). The cause of the polarized pattern of entry remains to be investigated. Previously published data have indicated that C6 transporters are involved in the entry of C6 ceramide into the cells (16). However, in the present study, the fluorescence signal was observed to occur between two dividing cells and we hypothesized that entry is likely to occur at mitosis initiation sites.

### Effect of inhibitors of lipid rafts/caveolae on C6 ceramide entry into ovarian cancer cells

**Figure 2:**
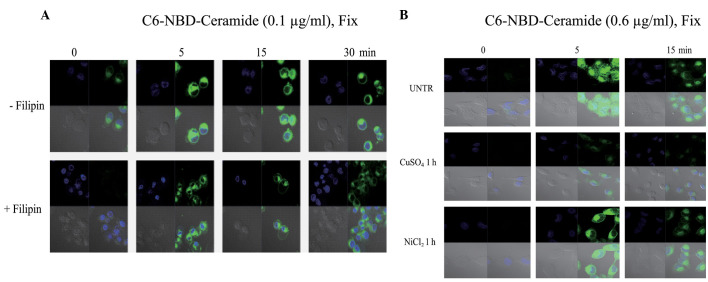
Effect of inhibitors on polarized entry of C6-NBD-ceramide into ovarian cancer cells. CaOV3 cells were cultured in 8-well chamber slides and treated with or without (A) a lipid raft/caveolae inhibitor or with (B) water channel inhibitors, CuSO_4_ or NiCl_2_. The cells were fixed with formaldehyde, processed and observed under a confocal microscope. Fluorescent short chain C6-NBD sphingomyelin, C6-NBD; Green, C6-NBD-ceramide; blue, nuclear staining.

Previous studies have indicated that the synergism of C6 ceramide and taxol is mediated by the inhibition of the EGF cell surface receptor and the ERK/AKT cell survival pathway, and that the action occurs in the initial hours following treatment. C6 ceramide is a membrane-permeable molecule that is currently hypothesized to enter the cells evenly through diffusion, although an accumulating number of studies have also demonstrated the existence of ceramide transporters (14–16). Our data also demonstrated that C6 ceramide rapidly enters cells in a polarized manner. Lipid rafts/caveolae have previously been identified as important for the process of signal transduction (19). To further investigate the entry of C6 ceramide, in the present study, the cells were pretreated with filipin, an inhibitor of lipid rafts/caveolae. Filipin inhibited the C6 ceramide entry into the CaOV3 cells (Fig. 2), indicating that lipid rafts/caveolae may be involved in the entry of C6 ceramide into the cells. Maintaining membrane structure or topology may potentiate the synergistic effect of taxol and ceramide on the apoptosis of cancer cells.

### Effect of water channel inhibitors on entry of C6 ceramide into ovarian cancer cells

Previous studies have demonstrated that molecules other than water may also enter the cells via water channels or aquaporins (20,21). Aquaporins also play critical roles in processes other than the transport of water (21,22). Our previous studies revealed that the EGFR-mediated expression of aquaporins is associated with cell migration in normal and cancer cells (23,24). To investigate whether C6 ceramide enters the cells via aquaporins or whether the entry is only partially associated with aquaporins, the inhibitors of aquaporins, CuSO_4_ and NiCl_2_, were utilized. The results revealed that CuSO_4_ inhibits C6 ceramide entry into the cells, but that NiCl_2_ does not (Fig. 2B), indicating that C6 ceramide may partially enter the cells via aquaporins.

### Effect of taxol and doxorubicin on the polarized entry of C6 ceramide into ovarian cancer cells

**Figure 3:**
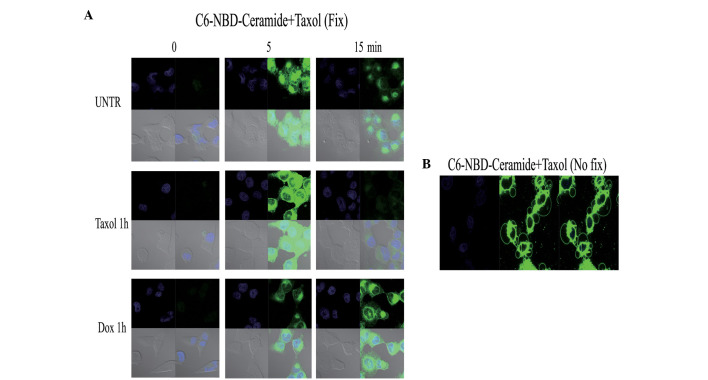
Effect of chemotherapeutic agents on C6-NBD-ceramide polarized entry into ovarian cancer cells. (A) CaOV3 cells were cultured in 8-well chamber slides, pretreated with taxol or doxorubicin for 1 h and then treated with C6-NBD-ceramide for 5 or 15 min. The cells were fixed with formaldehyde and processed for confocal microscopy. (B) The cells were pretreated with taxol and then C6-NBD-ceramide and processed for confocal microscopy without fixation. Fluorescent short chain C6-NBD sphingomyelin, C6-NBD; Green, C6-NBD-ceramide; blue, nuclear staining.

Paclitexal and doxorubicin have been successfully administered clinically in numerous cancer types (25). Our previous study demonstrated that together with C6 ceramide, taxol synergistically inhibits cell proliferation and cell migration (18). To further determine whether taxol affects C6 ceramide entry into ovarian cancer cells, in the present study, the cells were pretreated with taxol for 1 h and then treated with C6-NBD. The results indicated that taxol disrupted the polarized pattern of C6 entry. Notably, doxorubicin, a commonly utilized therapeutic agent, had no such effect (Fig. 3A). A previous study has shown that exogenous C6-ceramide induces endocytic vesicle formation and causes enlarged late endosomes and lysosomes in mouse fibroblasts (2). In the present study, the combination of C6-NBD and taxol was also observed to induce vesicle formation (Fig. 3B). The cause and effect of the formation of these vesicles requires further investigation, however, we hypothesize that vesicle formation may enhance apoptotic activity when using taxol and C6 ceramide in combination, as observed in our previous study (18).
